# Stability Analysis of Free Vibration of Gun Drill Rod

**DOI:** 10.3390/ma18061241

**Published:** 2025-03-11

**Authors:** Jingmin Ma, Wenli Yao

**Affiliations:** 1 College of Energy and Mining Engineering, Shandong University of Science and Technology, Qingdao 266590, China; skd992194@sdust.edu.cn; 2 School of Science, Qingdao University of Technology, Qingdao 266520, China

**Keywords:** gun drill rod, Rayleigh rotor, vibration stability, free vibration

## Abstract

Gun drills are widely utilized for deep hole machining due to their high aspect ratio, low stability, and susceptibility to vibrations, which can adversely affect machining accuracy and efficiency. During the machining process, cutting fluid must be injected through the drill rod to facilitate chip removal, resulting in a cross-sectional design with circumferential asymmetry. This asymmetry introduces more complex dynamic characteristics compared to conventional circular cross-section rotors. This study develops a Rayleigh rotor model of the gun drill rod and employs the Galerkin method to evaluate its free vibration characteristics and stability. The findings reveal that the gun drill rod exhibits distinct unstable speed ranges compared to circular cross-section rotors. Furthermore, the impact of various dimensional factors on the free vibration characteristics and stability of gun drill pipes was explored through detailed examination. The theoretical results were validated through comparative analysis with ANSYS simulations, confirming the reliability of the findings in this study.

## 1. Introduction

Rotors are fundamental components in mechanical systems, playing a critical role in determining the operational stability and efficiency of machinery [1]. Traditionally, rotors used in engineering applications are designed with solid or hollow circular cross-sections, which exhibit excellent stability due to their inherent circumferential symmetry [2]. However, in practical engineering scenarios, the design and implementation of rotors are often constrained by complex manufacturing processes, specific installation requirements, material property variations, and diverse operating conditions. These constraints frequently necessitate the use of rotors with circumferentially asymmetric cross-sections [3]. Examples of such rotors include gun drill rods and BTA (Boring and Trepanning Association) drill rods, which are widely employed in deep hole drilling operations [4,5,6,7]. These rods must simultaneously perform cutting operations, facilitate the flow of high-pressure cutting fluid into the cutting zone, and enable chip removal. The functional design of these rods inherently results in circumferentially asymmetric cross-sections. Specifically, in gun drill rods, the cutting fluid flows through the interior of the rod, while chips are discharged through external grooves. In contrast, in BTA drill rods, the cutting fluid flows through the annular gap between the rod and the workpiece, and chips are evacuated through internal channels within the rod.

The irregularity of such cross-sections introduces significant challenges in determining key mechanical parameters, including the moment of inertia, centroid position, and anisotropic material properties, which are relatively straightforward to compute for symmetric cross-sections. Consequently, researchers must account for these complexities during the modeling process to ensure that the model accurately captures the structural characteristics of the rotor, thereby facilitating reliable theoretical analysis and numerical simulations [8,9,10]. From a theoretical standpoint, the study of rotors with circumferentially asymmetric cross-sections extends the scope of traditional rotor dynamics, offering new insights that contribute to the advancement and enrichment of the theoretical framework for rotor vibration analysis.

While the dynamic characteristics of circumferentially symmetric rotors and rotor systems with various materials and structural configurations have been extensively investigated, research on the dynamic behavior of rotors with circumferentially asymmetric cross-sections remains relatively limited. This gap in the literature underscores the need for further exploration into the unique dynamic responses and stability characteristics of asymmetric rotors, which are increasingly prevalent in advanced engineering applications.

P. Jamshidi et al. [11] analyzed the nonlinear vibration in a rectangular cross-section rotor system using a multiscale method, demonstrating that asymmetry has a significant impact on the dynamic characteristics of the rotor system. Bavi R et al. [12] conducted dynamic analysis on composite laminated rotors, employing rectangular sections to simulate the geometric asymmetry of the cross-section, accounting for gyroscopic coupling effects, and utilizing Euler angles to achieve angular velocity. Their research encompassed multiscale analytical studies of parameter excitation systems under both resonant and non-resonant conditions. In deep hole machining, the substantial hole depth necessitates slender drill rod structures, resulting in low structural rigidity. The consequent vibration problem during the working process significantly affects machining efficiency and the surface quality of the hole. This has prompted extensive research interest in deep hole drilling, with primary focus areas including surface quality issues [13,14], vibration suppression and process optimization [15,16,17,18,19], and innovative shock-absorbing drill rod design [20,21,22]. However, studies on the stability of free vibration in drill pipes are relatively limited. Quanbin Zhang et al. [23] established a fluid-structure coupled transverse vibration differential equation for boring bars using Hamilton’s principle, taking into account the effects of bending, torsion, and axial deformation. The transverse vibration characteristics were solved through application of the Galerkin method. This model can be used to analyze the relationships between system stability and key parameters including equivalent stiffness, equivalent shear modulus, and axial force. L. Li et al. [24] developed a dual nonlinear damping model and explored its damping mechanisms under nonlinear stiffness. Bayly [25] compared theoretical predictions with experimental results, analyzed the torsional vibration of the drill pipe system, and provided a detail analysis of the distribution range of tool chatter frequencies and stability criteria, ultimately revealing significant nonlinear characteristics in the drill pipe system during deep hole machining. Smith and Johnson [26] investigated the vibration characteristics of asymmetric rotors under high-speed conditions, highlighting the significant influence of cross-sectional asymmetry on dynamic stability. Sabuncu and Evran [27] explored the dynamic stability of a rotating Timoshenko beam with an asymmetric cross-section under lateral parametric excitation, highlighting the critical role of geometric design in minimizing vibrations. Zhang et al. [28] investigated the nonlinear resonant responses of hyper-elastic cylindrical shells with initial geometric imperfections, demonstrating the importance of material properties and geometric nonlinearity in vibration stability. Zou et al. [29] studied the compression stability of a coupler under initial asymmetric conditions, providing insights into the effects of asymmetry on structural stability and vibration behavior. These studies collectively underscore the importance of addressing the unique challenges posed by asymmetric rotors and provide valuable insights for the design and optimization of such systems.

Previous dynamic analyses have typically employed either predetermined equivalent stiffness for drill pipes or modeled them as circumferentially symmetric circular or hollow circular section rods. This study takes the gun drill rod as an example for analysis and models it as a cantilever beam structure with an asymmetric cross-section. The research method encompasses the development of free vibration equations within a dynamic coordinate system, analysis of free vibration characteristics and stability parameters for asymmetric shafts, and validation through ANSYS finite element analysis, thereby establishing a relatively complete analytical system.

## 2. Materials and Methods

### 2.1. Mechanical Model

**Figure 1:**
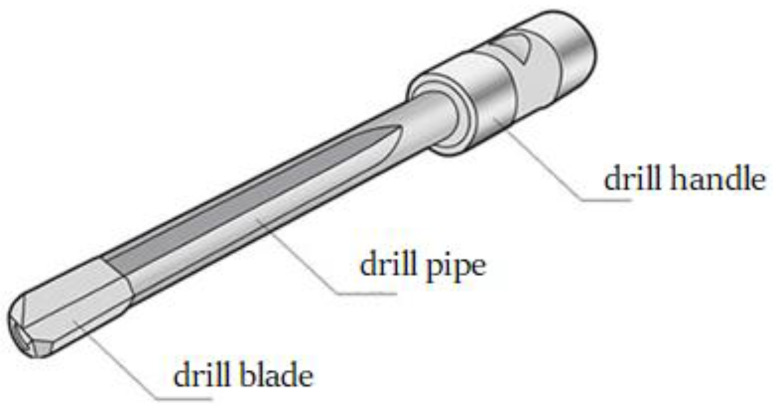
Structural diagram of gun drill assembly.

**Figure 2:**
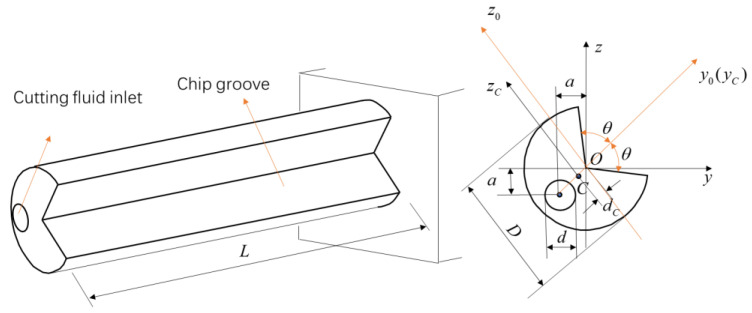
Simplified gun drill rod model.

The structure of the gun drill, as shown in Figure 1, consists of three main components: the drill blade, drill pipe, and drill handle. The drill handle serves as the connection point with machine tools, while the drill blade performs cutting operations. This study focuses on the analysis of the drill pipe section, disregarding the structural characteristics of the drill blade. For dynamic analysis, the drill pipe is simplified as an isotropic cantilever beam with a circumferentially asymmetric cross-section, as shown in Figure 2.

To facilitate analytical and computational efficiency, the drill pipe cross-section is assumed to have a symmetrical $y_{0}$-axis (or $y_{C}$-axis). The axes $y_{C}$ and $z_{C}$ represent the centroidal principal inertial axes of the section. The bending vibration of the drill rod predominantly occurs in the direction corresponding to the minimum moment of inertia, which correlates with minimum bending stiffness. Point *C* denotes the centroid of the cross-section, with the corresponding axes $y_{C}$ and $z_{C}$ serving as the principal inertial axes through this centroid. The cross-sectional area is designated as *A*, and the distance from the centroid to the coordinate origin is represented by $d_{C}$. *D* denotes the outer diameter of the gun drill, *a* signifies the location of the cutting fluid inlet position, *d* represents the size of the cutting fluid inlet hole, *θ* indicates the chip groove size dimensions, and *L* represents the length of the gun drill. Therefore, the principal moment of inertia through the centroid is defined as:

(1) $$I_{y_{C}}=I_{y_{0}}$$

(2) $$I_{z_{C}}=I_{z_{0}}-d_{C}^{2}A$$

where

$$I_{y_{0}}=\frac{\pi D^{4}}{64}-\frac{D^{4}(\theta -\sin\theta \cos\theta )}{64}-\frac{\pi d^{4}}{64}$$

$$I_{z_{0}}=\frac{\pi D^{4}}{64}-\frac{D^{4}(\theta +\sin\theta \cos\theta )}{64}-\frac{\pi d^{4}}{64}-\frac{\pi d^{2}a^{2}}{2}$$

$$A=\frac{\pi D^{2}}{4}-\frac{\theta D^{2}}{4}-\frac{\pi d^{2}}{4}$$

$$d_{C}=\frac{-D^{3}\sin\theta +3\sqrt{2}\pi ad^{2}}{3(\pi D^{2}-\theta D^{2}-\pi d^{2})}$$

### 2.2. Assumptions of the Rayleigh Rotor Model

The Rayleigh rotor model is employed in this study due to its ability to capture the essential dynamics of rotating structures while maintaining computational simplicity. The key assumptions of the model are as follows:

1. Small Displacements: The model assumes that the displacements of the rotor are small relative to its dimensions, allowing for linearization of the governing equations.
2. Isotropic Material: The drill pipe is assumed to be made of an isotropic material, meaning its mechanical properties are uniform in all directions.
3. Negligible Shear Deformation: The model neglects shear deformation, focusing primarily on bending vibrations.
4. No Damping: The initial analysis assumes no external damping; however, damping effects can be incorporated in subsequent studies.
5. Constant Rotational Speed: The rotor is assumed to rotate at a constant angular velocity, neglecting transient effects.
6. These assumptions simplify the analysis while still providing accurate insights into the dynamic behavior of the gun drill rod.

### 2.3. Vibration Equation of the Rayleigh Rotor for Gun Drill Rod

Based on the literature [26,30], the vibration equation of the Rayleigh rotor in a dynamic coordinate system is expressed as:

(3) $$EI_{y_{C}}v^{⁗}+\rho A(\ddot{v}-2\Omega \dot{w}-\Omega^{2}v)-\rho I_{y_{C}}{\ddot{v}}^{\prime \prime }=0$$

(4) $$EI_{z_{C}}w^{⁗}+\rho A(\ddot{w}-2\Omega \dot{v}-\Omega^{2}w)-\rho I_{z_{C}}{\ddot{w}}^{\prime \prime }=0$$

where v and w represent the displacement of the cross-sectional centroid along the $y_{C}$ and $z_{C}$ axes, respectively. Ω denotes the angular velocity of the drill rod rotation, and *E* and ρ represent the Elastic Modulus and density of the drill rod material, respectively. Comparing Equations (3) and (4), it is evident that $-2\rho A\Omega \dot{w}$ and $-2\rho A\Omega \dot{v}$ characterize the effects of Coriolis acceleration, while $-\rho A\Omega^{2}w$ and $-\rho A\Omega^{2}v$ represent the rotational inertia forces.

### 2.4. Justification of Galerkin Method

The Galerkin method is chosen for this study due to its effectiveness in solving partial differential equations (PDEs) that describe the vibration of continuous systems. The method is particularly well-suited for this problem for the following reasons:

1. Accuracy: The Galerkin method provides high accuracy in approximating the solutions of PDEs by projecting the problem onto a finite-dimensional subspace spanned by a set of basis functions.
2. Flexibility: The method allows for the use of various basis functions, enabling the capture of complex vibration modes in asymmetric rotors.
3. Efficiency: By transforming the PDEs into a system of ordinary differential equations (ODEs), the Galerkin method significantly reduces computational complexity while maintaining the essential dynamics of the system.
4. Validation: The method has been widely validated in rotor dynamics and structural vibration analysis, making it a reliable choice for this study.

For the cantilever shaft analysis, the bending deformation is expressed as the following formulation:

(5) $$v=\sum_{j=1}^{N}W_{j}(t)\psi_{j}(x),\;w=\sum_{j=1}^{N}V_{j}(t)\psi_{j}(x)$$

where $\psi_{j}(x)$ denotes the mode shape of bending deflection. The bending mode function of a standard uniform cantilever beam is defined as:

(6) $$\begin{array}{l} \cos(\beta_{j})\cos h(\beta_{j})=-1\;,\;\lambda_{j}=-\frac{\cos\beta_{j}+\cosh\beta_{j}}{\sin\beta_{j}+\sinh\beta_{j}}\;j=1,2,\cdots N\\ \psi_{j}=\cos(\frac{\beta_{j}x}{L})-\cosh(\frac{\beta_{j}x}{L})+\lambda_{j}(\sin(\frac{\beta_{j}x}{L})-\sinh(\frac{\beta_{j}x}{L})) \end{array}$$

Substituting Equations (5) and (6) into vibration differential Equations (3) and (4), and applying the Galerkin method with weighted integration of mode functions, a system of 2N ordinary differential equations is derived:

(7) $$K\left\{X\right\}+G\{\dot{X}\}+M\left\{\ddot{X}\right\}=0$$

Herein, **K** represents the stiffness matrix, which is a composite of the stiffness due to elastic deformation and that induced by rotation. **G** denotes the damping matrix resulting from the gyroscopic effect of rotation, and **M** stands for the mass matrix. X, $\dot{X}$, and $\ddot{X}$ correspond to the system’s coordinate array, velocity array, and acceleration array, respectively. Their expressions are delineated as follows:

$$K=\left[\begin{array}{cc} K11_{ij} & 0\\ 0 & K22_{ij} \end{array}\right]\;M=\left[\begin{array}{cc} M11_{ij} & 0\\ 0 & M22_{ij} \end{array}\right]\;G=\left[\begin{array}{cc} 0 & G12_{ij}\\ G21_{ij} & 0 \end{array}\right]$$

$$X={\left(\begin{array}{cc} \sum_{j=1}^{N}V_{j}(t) & \sum_{j=1}^{N}W_{j}(t) \end{array}\right)}^{T}$$

The matrix elements are defined as:

$$K11_{ij}=\int_{0}^{L}(EI_{y_{C}}\psi_{j}^{⁗}\psi_{i}-\rho A\Omega^{2}\psi_{j}\psi_{i})dx$$

$$K22_{ij}=\int_{0}^{L}(EI_{z_{C}}\psi_{j}^{⁗}\psi_{i}-\rho A\Omega^{2}\psi_{j}\psi_{i})dx$$

$$M11_{ij}=\int_{0}^{L}(\rho A\psi_{i}\psi_{j}-\rho I_{y_{C}}\psi_{i}^{\prime \prime }\psi_{j})dx$$

$$M22_{ij}=\int_{0}^{L}(\rho A\psi_{i}\psi_{j}-\rho I_{z_{C}}\psi_{i}^{\prime \prime }\psi_{j})dx$$

$$G12_{ij}=\int_{0}^{L}-2\rho A\Omega \psi_{i}\psi_{j}dx$$

$$G21_{ij}=-{G12}_{ij}=\int_{0}^{L}2\rho A\Omega \psi_{i}\psi_{j}dx$$

The solution of Equation (7) yields the free vibration characteristics of the circumferentially asymmetric cross-section gun drill rod. (Appendix A provides the MATLAB (version MATLAB R2014b) program code for calculating each element in the **K**, **M**, and **G** matrices, using K11 as an example).

## 3. Results

### 3.1. Reliability Analysis

**Figure 3:**
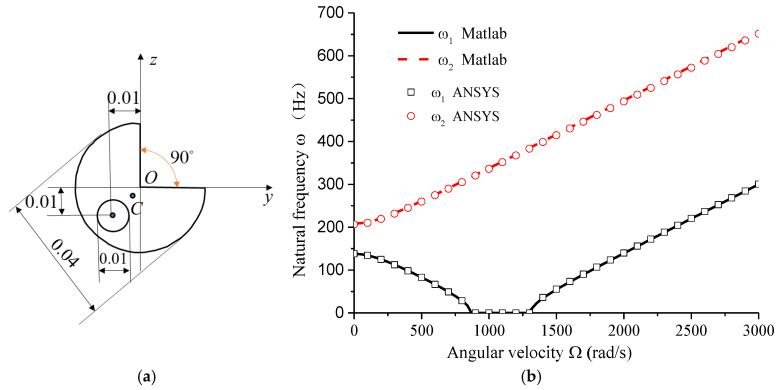
(**a**) The specific cross-sectional shape and dimensions of the gun drill rod (unit m); (**b**) comparison of natural frequency variation with rotational speed: MATLAB theoretical analysis vs. ANSYS analysis.

Gun drill rods are typically made of high-quality alloy steel, with the elastic modulus set to *E* = 210 GPa, *G* = 80 GP, and a material density of *ρ* = 7800 kg/m^3^. Analysis was conducted on a high-quality alloy steel drill rod with the following specifications: *L* = 0.4 m, *D* = 0.04 m, *d* = 0.01 m, *a* = 0.01 m, *θ* = 45° (shown in Figure 3a). The relationship between natural frequency and rotational speed is shown in Figure 3b.

As shown in Figure 3b, the natural frequencies in the dynamic coordinate system can be distinctly categorized into two types with increasing rotational speed: forward and backward motions. When the backward motion frequency approaches zero, the system becomes unstable. Due to the circumferential asymmetry of the rotor structure, the backward motion frequencies can reach zero or negative values within specific speed ranges (negative values are represented as zero in the plot). Figure 3 also compares the theoretical analysis results from this study with ANSYS (version ANSYS 19.0) simulation results, demonstrating a high degree of consistency between the two, thereby validating the reliability of the findings presented in this study. The ANSYS analysis model and the command stream for solving the natural frequencies considering the effects of rotation are provided in Appendix B.

The observed behavior of the natural frequencies highlights the unique dynamic characteristics of circumferentially asymmetric rotors. Unlike symmetric rotors, which typically exhibit a single critical speed, the gun drill rod demonstrates a range of unstable speeds where the backward motion frequency drops to zero. This phenomenon is directly linked to the asymmetric cross-section, which introduces additional complexities in the rotor’s dynamic response. The comparison with ANSYS simulations not only validates the theoretical model but also underscores the importance of considering cross-sectional asymmetry in rotor dynamics. The close agreement between the theoretical and simulation results suggests that the proposed model can accurately capture the dynamic behavior of asymmetric rotors, providing a reliable tool for further analysis and design optimization.

**Figure 4:**
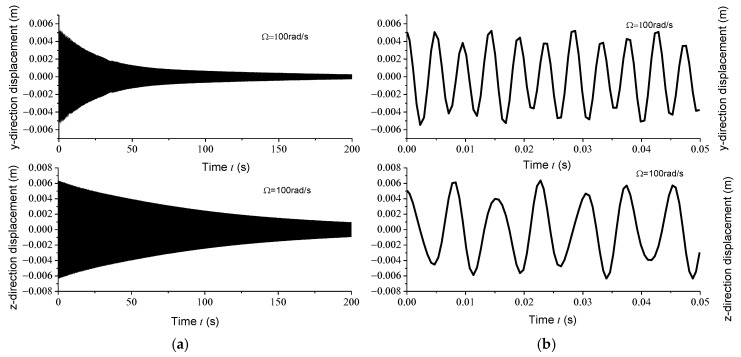
(**a**) Displacement responses in *y* and *z* directions at 100 rad/s within 200 s; (**b**) displacement responses in *y* and *z* directions at 100 rad/s within 0.05 s.

**Figure 5:**
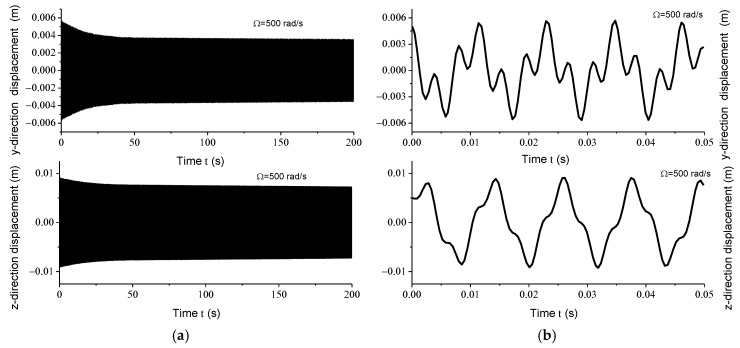
(**a**) Displacement responses in *y* and *z* directions at 500 rad/s within 200 s; (**b**) displacement responses in *y* and *z* directions at 500 rad/s within 0.05 s.

**Figure 6:**
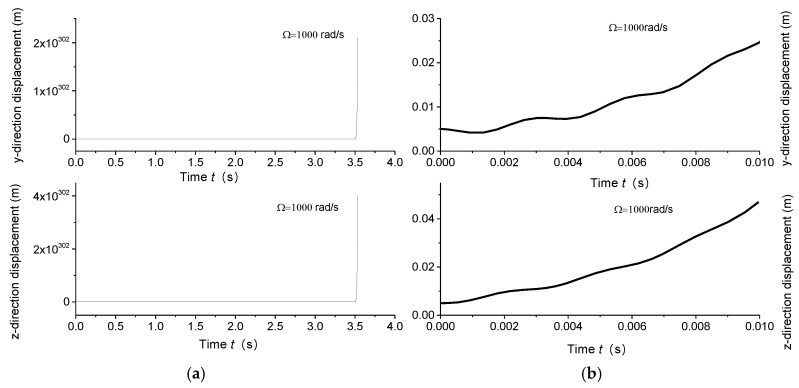
(**a**) Displacement responses in *y* and *z* directions at 1000 rad/s within 4 s; (**b**) displacement responses in *y* and *z* directions at 1000 rad/s within 0.01 s.

**Figure 7:**
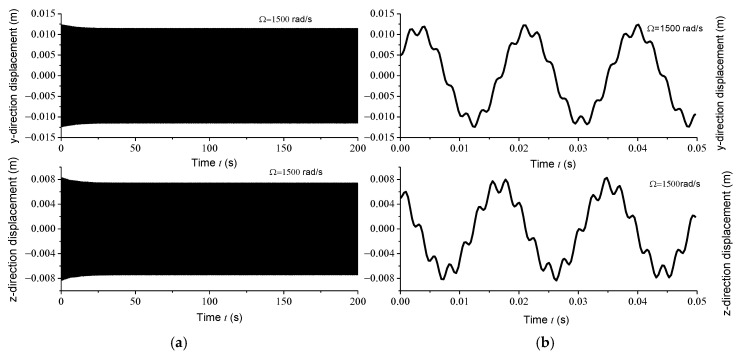
(**a**) Displacement responses in *y* and *z* directions at 1500 rad/s within 200 s; (**b**) displacement responses in *y* and *z* directions at 1500 rad/s within 0.05 s.

Displacement response analysis further confirms the existence of unstable regions. Due to the circumferential asymmetry of the rotor, the gun drill rod exhibits eccentricity. Under centrifugal force conditions, with specified initial displacement and velocity parameters, the Runge–Kutta method was implemented using the ODE45 function in MATLAB to analyze displacement responses at varying rotational speeds. Figure 4a, Figure 5a, Figure 6a and Figure 7a illustrate the displacement responses over a period of 0 to 200 s at different rotational speeds, while Figure 4b, Figure 5b, Figure 6b and Figure 7b provide magnified views of the displacement responses over shorter time intervals. Under identical initial conditions, the displacement response converges at rotational speeds of 100 rad/s, 500 rad/s, and 1500 rad/s. At lower speeds, the amplitude of the steady-state response is relatively small. However, as the speed increases, the inertial force intensifies proportionally, leading to increased steady-state response amplitudes. At Ω = 1000 rad/s, the displacement diverges rapidly, indicating the presence of a rotational speed instability region for the circumferentially asymmetric rotor.

### 3.2. Influence of Dimensional Factors on Vibration Stability

**Figure 8:**
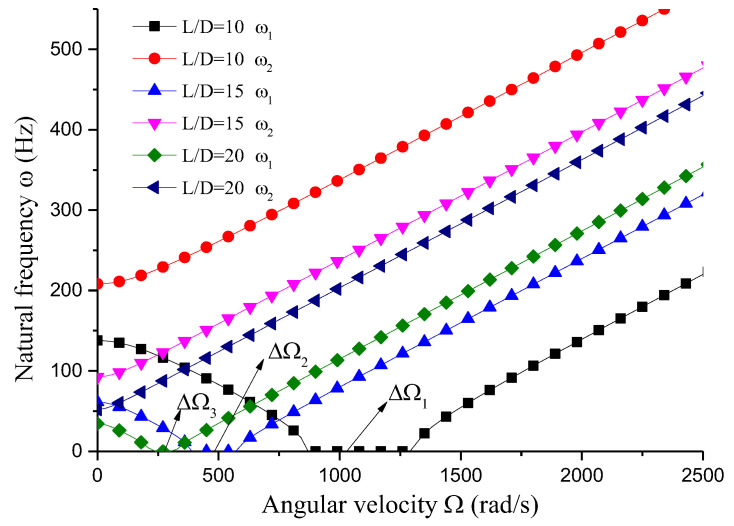
Influence of aspect ratio on vibration stability (*D* = 0.04 m, *a* = 0.01 m, *θ* = 45°, *d* = 0.01 m).

**Figure 9:**
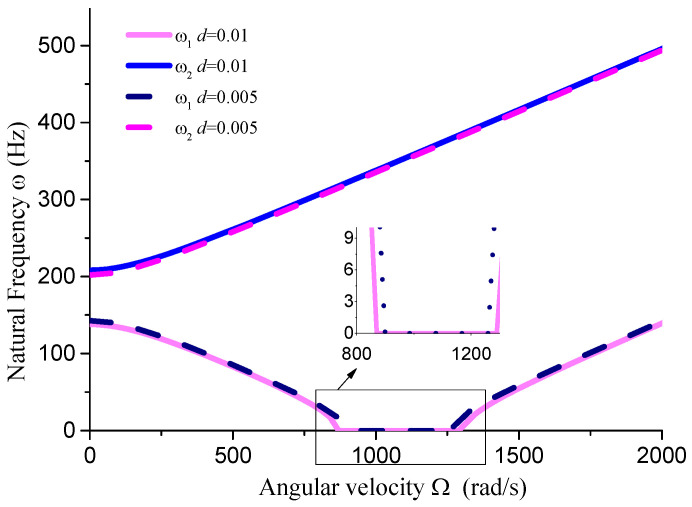
Influence of cutting fluid inlet dimensions on vibration stability (*L* = 0.4 m, *D* = 0.04 m, *a* = 0.01 m, *θ* = 45°).

**Figure 10:**
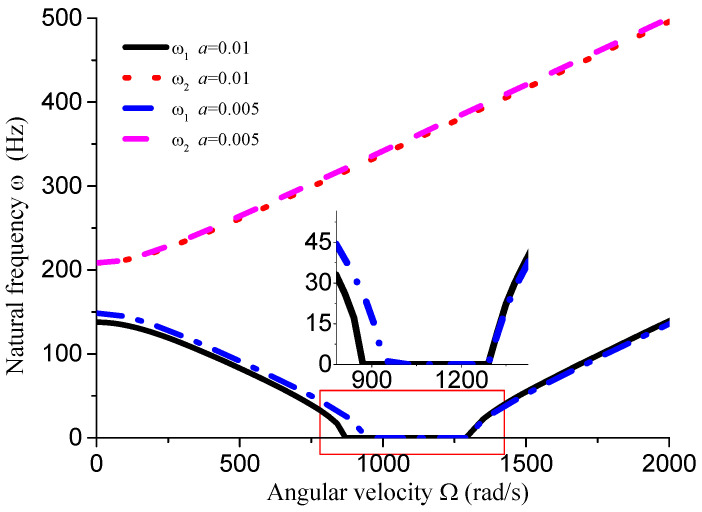
Influence of cutting fluid inlet position on vibration stability (*L* = 0.4 m, *D* = 0.04 m, *d* = 0.01 m, *θ* = 45°).

**Figure 11:**
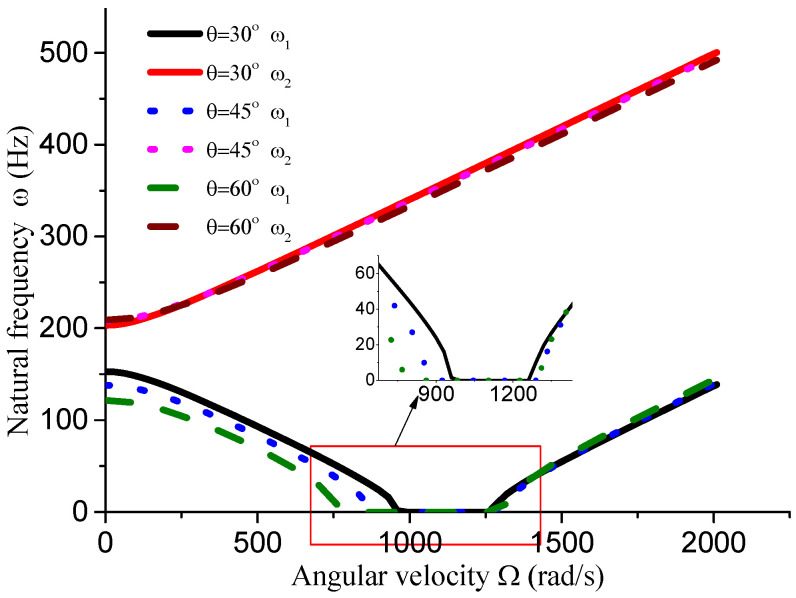
Influence of chip groove size dimension on vibration stability (*L* = 0.4 m, *D* = 0.04 m, *d* = 0.01 m, *a* = 0.01 m).

This section analyzes the influence of various dimensional parameters of the gun drill rod on its free vibration and stability. As shown in Figure 8, an increasing aspect ratio leads to a progressive decrease in both stiffness and natural frequency of the drill rod. Based on calculations, we obtain $\Delta \Omega_{1}=441$ rad/s, $\Delta \Omega_{2}=195$ rad/s, and $\Delta \Omega_{3}=14$ rad/s. The results indicate that as the aspect ratio increases and natural frequency decreases, the unstable region correspondingly diminishes. From Figure 8, Figure 9, Figure 10 and Figure 11 and Table 1, it can be observed that the dimensions of the cutting fluid inlet have a minimal impact on the natural frequency values. However, reducing dimensions of the cutting fluid inlet narrows the speed range of the unstable region (441 − 371 = 70 rad/s). Decreasing the distance from the cutting fluid inlet to the center of the outer circle only affects the stiffness of the gun drill along the $y_{C}$-axis, resulting in a variation in the first-order frequency. Conversely, reducing the distance between the cutting fluid inlet and the shaft increases the stiffness along the $y_{C}$-axis, leading to higher first-order natural frequency values while simultaneously narrowing the speed range of the unstable region (441 − 374 = 67 rad/s). The dimensions of the chip removal groove significantly influence the first-order frequency, while the second-order frequency remains relatively constant. Smaller chip groove dimensions correlate with higher first-order frequencies and reduced unstable regions (from 441 rad/s to 316 rad/s). Although increasing the dimensions of the chip removal groove enhances chip removal efficiency, it simultaneously introduces greater system instability.

The influence of dimensional factors on the vibration stability of the gun drill rod provides critical insights for the design and optimization of such systems. For instance, the aspect ratio plays a significant role in determining the stiffness and natural frequency of the rod. As the aspect ratio increases, the rod becomes more flexible, leading to lower natural frequencies and a reduced range of unstable speeds. This suggests that longer drill rods are more susceptible to instability at lower rotational speeds, which must be carefully considered during the design phase to avoid operational failures. The dimensions of the cutting fluid inlet, while having a minimal impact on the natural frequency, significantly affect the range of unstable speeds. Reducing the size of the cutting fluid inlet not only narrows the unstable region but also improves the overall stability of the system. This finding is particularly important for applications requiring high rotational speeds, as it allows for more stable operation without compromising the functionality of the drill rod. Similarly, the position of the cutting fluid inlet relative to the center of the rod influences the stiffness along the $y_{C}$-axis. Moving the inlet closer to the center increases the stiffness, resulting in higher first-order natural frequencies and a narrower unstable region. This indicates that the placement of the cutting fluid inlet can be optimized to enhance the stability of the drill rod, especially in high-speed applications. The dimensions of the chip removal groove also have a profound impact on the system’s stability. While larger grooves improve chip removal efficiency, they also reduce the first-order natural frequency and expand the unstable region. This trade-off between chip removal efficiency and system stability must be carefully balanced during the design process. On the other hand, smaller grooves increase the first-order frequency and reduce the unstable region, making them more suitable for applications where stability is a primary concern.

The analysis of dimensional factors reveals that the design of gun drill rods involves a complex interplay between various geometric parameters and their impact on vibration stability. By carefully optimizing these parameters, it is possible to achieve a balance between operational efficiency and system stability. The findings of this study provide valuable guidance for the design of gun drill rods and other circumferentially asymmetric rotors, highlighting the importance of considering cross-sectional asymmetry in rotor dynamics.

## 4. Conclusions

This study establishes a Rayleigh rotor model for a circumferentially asymmetric gun drill rod and analyzes its free vibration characteristics and rotational stability. The analysis reveals that, unlike rotors with well-defined critical speeds, the circumferentially asymmetric gun drill rod exhibits a distinct range of unstable rotational speeds within which displacement rapidly diverges. Investigation of the influence of dimensional factors on vibration stability yields the following conclusions: (1) An increase in the aspect ratio and natural frequency corresponds to an expansion of unstable regions. (2) Reducing the dimensions of the cutting fluid inlet effectively narrows the speed range of unstable regions. (3) Positioning the cutting fluid inlet closer to the coordinate origin increases the first-order natural frequency values and reduces the speed range of unstable regions. (4) Increasing the chip removal groove dimensions leads to a significant reduction in the first-order frequency and expansion of the speed range of unstable regions. The modeling method and conclusions presented in this study are applicable to the analysis of other circumferentially asymmetric rotors, which can provide valuable engineering guidance for the design of gun drill rods.

## Figures and Tables

**Table 1:** Influence of dimensional parameters on vibration stability.

Dimensional Parameters	ω_1_ (Hz, Ω = 0)	ω_2_ (Hz, Ω = 0)	∆Ω (rad/s)
*d* = 0.01 m *a* = 0.01 m θ = 45° *L* = 0.4 m	137.9	208.3	441
*d* = 0.005 m *a* = 0.01 m θ = 45° *L* = 0.4 m	142.7	202	371
*d* = 0.01 m *a* = 0.005 m θ = 45° *L* = 0.4 m	148.53	208.3	374
*d* = 0.01 m *a* = 0.01 m θ = 30° *L* = 0.4 m	152.85	203.35	316

**Figure A1:**
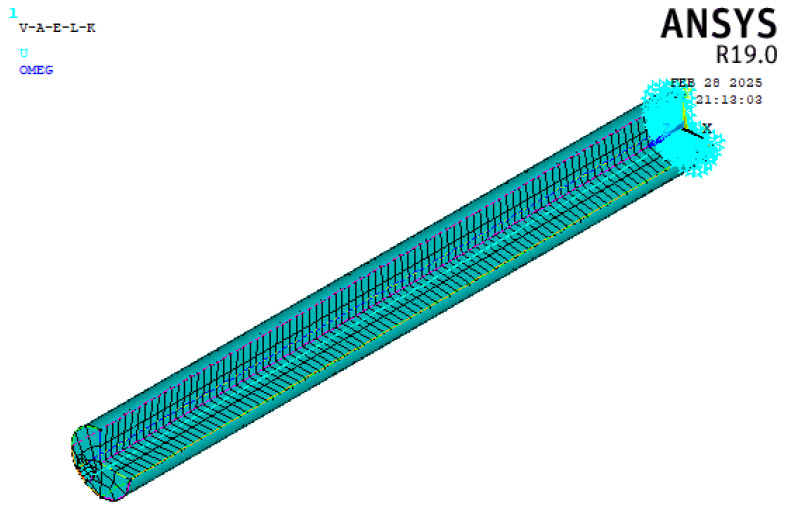
ANSYS analysis model.

## Data Availability

The original contributions presented in this study are included in the article. Further inquiries can be directed to the corresponding author.

## Appendix A

In MATLAB R14b, the program for calculating the stiffness matrix elements using the Galerkin method for bending vibration is as follows:

syms i j x

betajj = zeros(1,6);

betajj(1) = 1.8751040687119611138911068337620;

betajj(2) = 4.6940911329741741297993939951994;

betajj(3) = 7.8547574382376126322924392297864;

betajj(4) = 10.995540734875465460618215729482;

betajj(5) = 14.137168391046470716787553101312;

betajj(6) = 17.278759532088237449443113291636;

ii = 1;

omega = 500;

k11 = zeros(nj,nj);

for i = 1:nj

  for j = 1:nj

    betai = betajj(i);

    lambdai = −(cos(betai) + cosh(betai))/(sin(betai) + sinh(betai));

    phii = cos(betai*x/L)-cosh(betai*x/L) + lambdai*(sin(betai*x/L) − sinh(betai*x/L));

    betaj = betajj(j);

    lambdaj = −(cos(betaj) + cosh(betaj))/(sin(betaj) + sinh(betaj));

    phij = cos(betaj*x/L) − cosh(betaj*x/L) + lambdaj*(sin(betaj*x/L) − sinh(betaj*x/L));

    k11ijx1 = E*Iy*diff(phii,4)*phij − rho*A*omega^2*phii*phij;

    k11ijx = inline(vectorize(k11ijx1),‘x’,‘i’,‘j’);

    k11(i,j) = quadl(k11ijx,0,L,[],[],i,j);

  end

end

The K11 calculation process is also applicable to the calculation of individual elements in the mass matrix and damping matrix.

## Appendix B

### Appendix B.1

A three-dimensional rotor model using the SOLID186 element in ANSYS is illustrated in Figure A1, with one end free, the other end fixed, and subjected to inertial forces from rotation about its own axis.

### Appendix B.2

The following provides the command stream for natural frequency analysis in ANSYS considering rotational effects.

/PREP7

OMEGA,0,0,300,

CORIOLIS,1, , ,0,0

FINISH

/SOL

!*

ANTYPE,0

PSTRES,1

/STATUS,SOLU

SOLVE

FINISH

/POST1

FINISH

/SOL

!*

ANTYPE,2

!*

MODOPT,QRDAMP,8

MXPAND,2, , ,0

LUMPM,0

PSTRES,1

!*

MODOPT,QRDAMP,8,0,300,0,OFF

/STATUS,SOLU

SOLVE

FINISH

/POST1

SET,LIST
